# Risk factors for surgical site infection after groin hernia repair: does the mesh or technique matter?

**DOI:** 10.1007/s10029-021-02512-7

**Published:** 2021-10-01

**Authors:** N. Christou, F. Ris, D. Naumann, J. Robert-Yap, M. Mathonnet, J.-F. Gillion, The Club-Hernie Members

**Affiliations:** 1 Service de Chirurgie Digestive, Endocrinienne et Générale, CHU de Limoges, Avenue Martin Luther King, 87042 Limoges Cedex, France; 2 Department of Visceral Surgery, Geneva University Hospitals and Medical School, Rue Gabrielle Perret, 4, 1205 Geneva, Switzerland; 3 University Hospitals Birmingham NHS Foundation Trust, Birmingham, B152TH UK; 4 Unité de Chirurgie Viscérale Et Digestive, Hôpital Privé d’Antony, 1, Rue Velpeau, 92160 Antony, France

**Keywords:** Surgical site infection, Groin hernia repair, Risk factors

## Abstract

**Introduction:**

Surgical site infections (SSIs) following groin hernia repair (GHR) are getting rarer in high income countries despite a wider use of meshes. Among the risk factors for SSIs, those related to the mesh and the surgical technique have rarely been described.

**Methods:**

A registry-based multicenter study using prospectively collected data, including SSIs and their potential risk factors, was conducted in the French Hernia-Club.

**Results:**

Between 2012 and 2019, 21,976 consecutive unselected adult patients aged 64.8  ±  15.4 years old (88.9% male) underwent GHR (83.5% unilateral). Fifty four percent were laparoscopic; 97.6% used mesh. The overall incidence of SSI was 0.26%. The incidence of SSI was respectively, 0.24% and 0.19% (*p*  =  0.420) in open vs laparoscopic repairs; 0.19% and 0.25% (*p*  =  0.638) for polyester vs polypropylene mesh; In adjusted multivariate analysis focusing on macroporous meshes (which were the most implanted meshes: 23,148 out of 24,099), there were no differences in terms of SSIs’ rates regarding the technique: open versus laparoscopy (*p*  =  0.762) nor the type of mesh used: polypropylene versus polyester (*p*  =  0.557).

**Conclusion:**

The rate of SSI following GHR was low in this large registry study. Mesh type and surgical technique did not affect SSIs rates. Caution is advised when interpreting these data due to this very low rate of SSI and the potential for a type II error.

**Supplementary Information:**

The online version contains supplementary material available at 10.1007/s10029-021-02512-7.

## Introduction

Groin hernia repair (GHR) is the most frequent intervention in elective general surgery. It represents around 2000 operations per million people per year in High Income Countries [1].

According to systematic reviews [1]^2^ updated in the Herniasurge guidelines (Herniasurge) [3] the rate of surgical site infections (SSIs) after GHR broadly varies across the studies from 1.6% in low-risk environment to 4.2% [4] in high-risk environment. Recently published rates are even lower: 0.4% in the US NSQIP (National Surgery Quality Improvement Program) [5]; 0.64% decreasing to 0.2% after laparoscopic GHR [6].

Numerous risk factors for SSIs after (elective) GHR (such as diabetes, current smoking) have already been identified [5]. Among the risk factors for SSIs, those potentially related to the type of mesh and the surgical technique used have rarely been studied.

Hence, the aim of this study, was to focus on type of mesh and surgical technique as potential risk factors for SSI after elective GHR.

## Materials and methods

### Study design

We conducted an observational cohort study using a prospective database (close ended input boxes) of patients who had GHR during a 7-year period from different French centres. This database included all patients over the age of 16 years old who were operated on from GHR in the French Hernia-Club registry between 2012 and 2019. The patients were informed that their anonymous data were registered and that they would receive a phone questionnaire at different stages of their follow-up. Only the operating surgeon and the CRA were able to link the randomly allocated identifying number and the patient. The data were stored in a specialized Swiss data bank where they were protected against network intrusion. The registry complies with the requirements of the General Data Protection Regulation (GDPR), the French ‘Méthodologies de référence de la Commission Nationale Informatique et Liberté’ (MR001, MR003) and the different specific French ethics committees. STROBE (Strengthening the Reporting of Observational studies in Epidemiology) [5] and the European Registry of Abdominal Wall Hernias (EuraHS) recommendations [6] were used for the conduct and reporting of our study.

### Club-Hernie registry

The French “Club Hernie” is composed of surgeons specially interested in hernia repairs. Hernia club registry is composed of comprehensive anonymous patient data as a registered part of the operating surgeons’ office files [not in the online database, to comply with the European General Data Protection Regulation (GDPR)]. All patients’ anonymous data are registered in the online database, in real time, consecutively, without selection. The comprehensiveness is ensured with a signed quality charter and checked with audit-reconciliations between the entries in the database and the administrative parietal activity records of the member. Completion gauges, updated with every input, help the surgeon to ensure the comprehensiveness of data.

### Patient follow up

Clinical follow-up was performed by the operating surgeon at discharge and at the first month clinical visit. In case of any symptoms, an additional visit was scheduled between the 3rd and the 6th month post-operative check. The follow-up at 2 years (2Y-Fu) and 5 years (5Y-Fu) consisted of a telephone interview following a validated phone questionnaire performed by an independent clinical research assistant (CRA) blinded to the surgical procedure. The wording of the questionnaire used layperson terminology and the four level VRS (Verbal Rating Scale). Answers were recorded verbatim, without any medical correction according to our Patient Reported Outcome Measures (PROM) policy. In case of any reported event, the patient was strongly recommended to schedule a clinical visit. A retro-control of the registered outcomes was done during the phone interview. In case of discrepancy, the medical chart was reviewed with the operating surgeon. Patients were considered lost to follow-up after five failed attempts to contact them on different days. Patients who decline to participate in the telephone interview were considered lost to follow-up but also recorded apart as potentially poor outcomes.

### Data collection

Data extracted from the registry included pre-operative data with patient characteristics, factors influencing wound healing or dissection, surgical history and hernia characteristics. Operative characteristics were also described, and included: open or laparoscopic; surgical technique; use of mesh; technique of mesh placement; mesh characteristics; duration of operation; and management of nerve dissection (preservation of nerves, section with buried or coagulated ends). Post-operative outcomes occurring in the first 30 days after the surgery were collected. Complications were graded according to the Clavien–Dindo classification. At every follow-up, a quality of life (QoL) questionnaire and a patient self-assessment of the surgery (PROM) form were filled out and registered in the database.

### Outcomes

The primary outcome measure was the occurrence of any SSI within 1 year of postoperative follow-up. These infections were detected at regular clinical controls at discharge, at the 1-month post-operative clinical visits and during any additional visits in case of any symptoms during the 1-year postoperative period. SSI was defined following the Centers for Disease Control and Prevention criteria [7]. SSIs encompassed superficial, deep, and organ space infections. More precisely, we included patients with either non peri-mesh infected collection (subcutaneous) or peri-mesh infected collection. We also recorded whether SSI was managed with mesh conservation or with mesh explant.

### Data analysis

Categorical data are summarized as number and percentage, and normal continuous data are summarized as mean and standard deviation (±  SD). Comparisons were conducted using Chi-square tests (or Fisher exact tests if the conditions to apply Chi-square were not verified by data). The difference in risk of SSI between polyester and polypropylene prosthesis was assessed and reported with an exact 95% confidence interval (95% CI). Adjusted associations with the risk of SSI were investigated with a multiple logistic regression model. Due to the low number of SSIs, the number of independent variables was limited to five. These independent variables were selected a priori and no statistical procedure of variable selection was used. Unadjusted and adjusted odds ratios were reported with their 95% confidence intervals. The type I error was 0.05 two-sided for all statistical analyses. Analyses were conducted with software R version 4.0.2 (Foundation for Statistical Computing, Vienna, Austria) using the R package Desctools [Andri Signorell et mult. al. (2020). DescTools: tools for descriptive statistics, R package version 0.99.38].

## Results

### Patients’ characteristics

During the study period (June 2011–May 2019), 25,499 patients underwent GHR in elective surgery conditions. 86 surgeons participated in this study. After exclusion of patients with missing data, there were 21,976 patients who had 25,593 GHR (accounting for patients who had bilateral surgery). The mean age was 64.8 years old (±  15.4) years old, the majority of whom were male (88.9%). Of the 25,593 groin hernias, 24,076 were repaired with mesh and 589 without.

### Surgical site infections

Surgical site infections (SSIs) were identified in 57 patients (0.26%) within the study cohort. The groups with and without SSIs were comparable in terms of demographic characteristics (Table 1a, b) and surgical characteristics (Table 2a). There was no statistically significant difference between the two groups with SSIs or without, regarding the operated side (left or right), the operated hernia recurrent or primary, the technique used (laparoscopic or laparotomy and with or without a mesh), the management of nerves or the use of naropeine (ropivacaine) infiltration (Table 2a). Similarly, there was no significant difference between the two groups with or without SSI when a mesh was implanted regardless of the surgical characteristics already described above, the mesh characteristics, or the type of fixation, Table 2b.

**Table 1:** Patients’ characteristics

a: Patients characteristics—general demography			
	All patients (*n* = 21,976)	No SSI (*n* = 21,919)	SSI (*n* = 57)
Bilateral	3617 (16.5%)	3607 (16.5%)	10 (17.5%)
Unilateral	18,359 (83.5%)	18,312 (83.5%)	47 (82.5%)
Gender			
Female	2446 (11.1%)	2440 (11.1%)	6 (10.5%)
Male	19,530 (88.9%)	19,479 (88.9%)	51 (89.5%)
Age, years	64.8 (15.4)	64.8 (15.4)	63.5 (16.8)
BMI	24.9 (3.4)	24.9 (3.4)	25.5 (3.2)
ASA score			
I	10,609 (49%)	10,583 (49%)	26 (48.1%)
II	8049 (37.2%)	8026 (37.1%)	23 (42.6%)
III	2962 (13.7%)	2957 (13.7%)	5 (9.3%)
IV	39 (0.2%)	39 (0.2%)	0 (0%)
V	2 (0%)	2 (0%)	0 (0%)
Abdominal pressure	5964 (28.1%)	5950 (28.1%)	14 (25.5%)
Smoking	3213 (15.2%)	3206 (15.2%)	7 (13.2%)
Physical effort	7130 (34.2%)	7114 (34.2%)	16 (28.6%)
Dissection factors	6791 (32.2%)	6777 (32.3%)	14 (26.4%)

b: Patients’ characteristics—wound healing factors			
	All patients (*n* = 21,976)	No SSI (*n* = 21,919)	SSI (*n* = 57)
Diabetes	1069 (4.3%)	1066 (4.3%)	3 (5.5%)
Corticotherapy		325 (1.3%)	323 (1.3%)
Pelvic radiotherapy	305 (1.2%)	305 (1.2%)	0 (0.0%)
Chemotherapy	0 (0.0%)	0 (0.0%)	0 (0.0%)
Anticoagulants	3339 (13.6%)	3331 (13.6%)	8 (14.5%)

**Table 2:** Surgical characteristics

a: General surgical characteristics			
	All sides (*n* = 25,593)	No SSI (*n* = 25,536)	SSI (*n* = 57)
Side			
Left	11,807 (46.1%)	11,780 (46.2%)	27 (47.4%)
Right	13,786 (53.9%)	13,756 (53.8%)	30 (52.6%)
Recurrences	1754 (7.1%)	1750 (7.1%)	4 (7.4%)
Technique			
Without mesh	589 (2.4%)	587 (2.4%)	2 (3.7%)
Laparoscopy	13,343 (54.0%)	13,317 (54.1%)	26 (48.1%)
Open	10,756 (43.6%)	10,730 (43.6%)	26 (48.1%)
Nerve preservation	24,401 (99.7%)	24,348 (99.7%)	53 (100.0%)
Naropeine infiltration	15,279 (62.6%)	15,241 (62.6%)	38 (71.7%)

b: Surgical characteristics of sides with a mesh			
	Sides with a mesh (*n* = 24,099)	No SSI (*n* = 24,047)	SSI (*n* = 52)
Side			
Left	11,134 (46.2%)	11,109 (46.2%)	25 (48.1%)
Right	12,965 (53.8%)	12,938 (53.8%)	27 (51.9%)
Recurrence	1631 (6.9%)	1628 (6.9%)	3 (5.9%)
Technique			
Laparoscopy	13,343 (55.4%)	13,317 (55.4%)	26 (50.0%)
Open	10,756 (44.6%)	10,730 (44.6%)	26 (50.0%)
Mesh type			
Non-resorbable polyester	9678 (40.2%)	9660 (40.2%)	18 (34.6%)
Non-resorbable polypropylene	13,310 (55.2%)	13,277 (55.2%)	33 (63.5%)
Non-resorbable PTFE	0 (0.0%)	0 (0.0%)	0 (0.0%)
Non-resorbable type undefined	826 (3.4%)	825 (3.4%)	1 (1.9%)
Resorbable synthetique	7 (< 0.1%)	7 (< 0.1%)	0 (0.0%)
Porosity			
Microporous	673 (2.8%)	673 (2.8%)	0 (0.0%)
Macroporous	23,148 (97.2%)	23,096 (97.2%)	52 (100.0%)
Fixation			
No	13,980 (58.4%)	13,954 (58.5%)	26 (63.4%)
Yes—mixte	3071 (12.8%)	3064 (12.8%)	7 (17.1%)
Yes—non-resorbable	1916 (8.0%)	1909 (8.0%)	7 (17.1%)
Yes—resorbable	4953 (20.7%)	4942 (20.7%)	11 (26.8%)

### Risk factors for SSI

#### Univariate analysis

Results of univariate analysis are shown in Tables 3. Neither factors linked to the patients’ characteristics nor factors linked to wound healing (Table 3a) were independently associated with SSI. Considering surgical factors (Table 3b), none were independently associated with SSI. Furthermore, focusing on sides with a mesh, no factors linked to surgery such as recurrences, technique used (open or laparoscopy), or mesh characteristics were associated with SSI occurrence (*p * =  0.638 for mesh type and *p*  =  0.406 for mesh porosity) (Table S1). Noticeably, no statistically significant difference in the SSI rate was observed between macroporous polyester vs polypropylene GHR (Table 3c). More precisely, the difference between polyester and polypropylene (both non-resorbable prostheses) was − 0.06% (− 0.18–0.06, 95% CI) (*p*  =  0.395) (Table 3c). The method of fixation of the mesh, nerve preservation and naropeine infiltration were not significantly linked to higher SSI rates (Table S1).

**Table 3:** Univariate analysis—risk factors of SSIs: patients and surgical factors

a: Patient factors (*n* = 21,976 patients)	
	Risk of SSI
Bilateral	*p* = 0.966
Bilateral	10/3617 (0.28%)
Unilateral	47/18359 (0.26%)
Gender	*p* > 0.99
Women	6/2446 (0.25%)
Men	51/19530 (0.26%)
Age, years	*p* = 0.305
16–54	16/5308 (0.30%)
55–64	7/4563 (0.15%)
65–74	14/6011 (0.23%)
75–and +	20/6094 (0.33%)
BMI	p = 0.666
Less than 20	2/1147 (0.17%)
20–25	25/10805 (0.23%)
26–30	25/8317 (0.30%)
31 and +	5/1454 (0.34%)
ASA score	*p* = 0.531
I	26/10609 (0.25%)
II	23/8049 (0.29%)
III–V	5/3003 (0.17%)
Abdominal pressure	*p* = 0.771
No	41/15241 (0.27%)
Yes	14/5964 (0.23%)
Smoking	*p* = 0.834
No	46/17953 (0.26%)
Yes	7/3213 (0.22%)
Physical effort	*p* = 0.456
No	40/13730 (0.29%)
Yes	16/7130 (0.22%)
Wound healing factors	*p* = 0.774
No	42/16605 (0.25%)
Yes	13/4454 (0.29%)

b: Surgical factors (*n* = 25,593 sides)	
	Risk of SSI
Recurrences	*p* = 0.792
No	50/22988 (0.22%)
Yes	4/1754 (0.23%)
Technique	*p* = 0.420
No mesh	2/589 (0.33%)
Laparoscopy	26/13343(0.19%)
Open	26/10756(0.24%)
Dissection factors	*p* = 0.446
No	39/14271 (0.27%)
Yes	14/6791 (0.21%)
Nerve preservation	*p* > 0.99
No	0/75 (0.00%)
Yes	53/24401 (0.22%)
Naropeine infiltration	*p* = 0.219
No	15/9128 (0.16%)
Yes	38/15279 (0.25%)
Side	*p* = 0.957
Left	27/11807 (0.23%)
Right	30/13786 (0.22%)

#### Multivariate analysis

Regardless of the association between technique used (laparoscopy or open) and the type of hernia operated (uni or bilateral), there was no significant difference in SSI number (*p*  =  0.267) After logistic regression adjusted for ASA score, bilateral hernia surgery or open surgery, none of these factors were linked to an increased risk of SSI (Table 4a). Further multivariate analysis was performed on the macroporous mesh group (number of sides analyzed  =  23,141). After adjustment of BMI, smoking status, ropivacaine infiltration and surgical technique used, there was no difference in SSI risk between each type of macroporous mesh used, especially between polyester and polypropylene meshes (*p*  =  0.557) (Table 4b).

**Table 4:** Multivariate analysis

a: Model 1 all sides (*n* = 25,536), logistic regression adjusted for ASA score^a^		
	OR (95%CI)	*p* values
Bilateral		
No (unilateral)	1 (reference)	
Yes (bilateral)	0.62 (0.29–1.21)	0.181
Technique		
Coelio	1 (reference)	0.901
Laparo	1.12 (0.63–1.98)	0.703
Pas de prothèse	0.83 (0.05–3.97)	0.855
ASA score		
I–II	1 (reference)	
III–V	0.66 (0.23–1.52)	0.384

b: Model 2—sides with a prosthesis « macroporeux»						
	*n* sides	Risk SSI	Unadjusted		Adjusted	
			OR (95% CI)	*p* values	OR (95% CI)	*p* values
BMI						
Less than 25	12,348 (53.9%)	24 (0.19%)	1 (reference)	0.531	1 (reference)	0.549
25–30	8994 (39.3%)	24 (0.27%)	1.37 (0.78–2.43)	0.272	1.38 (0.77–2.47)	0.279
31 and +	1554 (6.8%)	4 (0.26%)	1.33 (0.39–3.43)	0.603	1.04 (0.25–3.01)	0.944
Technique						
Laparoscopy	12,656 (54.7%)	26 (0.21%)	1 (reference)		1 (reference)	
Open	10,485 (45.3%)	26 (0.25%)	1.21 (0.70–2.09)	0.497	1.10 (0.59–2.11)	0.762
Mesh type						
Non resorbable polyester	9678 (41.8%)	18 (0.19%)	1 (reference)	0.497	1 (reference)	0.651
Non resorbable polypropylene	12,637 (54.6%)	33 (0.26%)	1.41 (0.80–2.55)	0.246	1.21 (0.65–2.31)	0.557
Naropeine infiltration						
No	8266 (36.2%)	14 (0.17%)	1 (reference)		1 (reference)	
Yes	14,542 (63.8%)	36 (0.25%)	1.46 (0.81–2.81)	0.228	1.34 (0.68–2.77)	0.408
Smoking						
No	19,009 (84.6%)	43 (0.23%)	1 (reference)		1 (reference)	
Yes	3457 (15.4%)	7 (0.20%)	0.89 (0.37–1.87)	0.786	0.95 (0.39–1.99)	0.898

^a^Interaction term between bilateral and technique was not statistically significant (*p*  =  0.388)

## Discussion

We report an overall 0.26% rate of SSI (by patient and by operated side) for a cohort of 21,976 patients having undergone an elective GHR and registered in the French Hernia club registry. They were considered as elective procedures (24,076 sides with a mesh and 589 sides without mesh), and classified as “clean surgery” without any bowel contamination (Altemeier Class 1). To our knowledge, this is the largest study of elective groin hernia repairs that investigates post-operative infection rates and SSIs risk factors potentially related to both mesh and surgical technique used including both laparoscopic and open procedures. Indeed, a recent study only explored the demographics and the preoperative risk factors (and encompassed emergency GHR) [6], another one compared, as second endpoint, the incidence of SSIs between laparoscopic and Lichtenstein techniques within a male cohort only [8].

Interestingly, in our study, after adjusted multivariate analysis, we didn’t highlight any risk factors of SSIs regarding both mesh type and technique (open versus laparoscopy) used.

The main advantage of our large French cohort, was that we were able to investigate which mesh characteristics might impact on SSI rates. Our work demonstrated no specific mesh features impact on SSI risk. No statistically significant difference in the SSI rate was observed between macroporous polyester vs polypropylene GHR, just a trend favouring polyester after adjusted multivariate analysis (Table 4b).

The recent cohort of 54,951 cases from the American College of Surgery National Surgical Quality Improvement Program (ACS NSQIP) reported by Sereysky et al. [5] focused on elective open repairs only. No risk factors for SSIs were found in both univariate and multivariate analyses of 18 parameters related to patients, surgery, or operative characteristics. These divergences of results from Sereysky et al. with our study, may be explained by the differences regarding (i) the methodology: only open repairs were included in the cohort of Sereysky et al. whereas we integrated open and laparoscopic repairs in our study, (ii) the characteristics of the cohort: percentage of patients within the American cohort with general risk factors of infections such as type 2 diabetes, obesity, current smoking, use of steroids, was higher.

In another large study on 17,388 patients from the Herniamed database [8], the SSI rates were statistically different (0.06% vs. 0.26%; *p*  =  0.003) in total extra peritoneal repair (TEP repair- by laparoscopy) and Lichtenstein technique (open). In our present study, the incidence of SSI (0.19% vs 0.24%; *p*  =  0.486) was not significantly different in the open vs. laparoscopic group. This difference may be lightened by the fact that in the French Hernia Club registry, Lichtenstein procedures are performed by specialists of this technique.

From an international point of view, ours is the first study to our knowledge to investigate the number of SSIs in elective surgery (without emergencies [9]). Previous data that examined SSI following elective GHR were derived from investigation of the merits of antibiotic prophylaxis [10]. SSI rates after GHR were reported as between 2.4 and 6%, which is higher than the current data. This may be explained by the fact that our database grouped high volume centres (around 150 GHR per year per surgeon). In France, since 2014, the French National monitoring program of surgical site infections (SSI) (called ISO-RAISIN) [11] has shown a steady and significant increase of the number of SSIs after GHR: 0.67% in 2013 versus 0.93% in 2017. These figures are threefold higher than our results. This difference may be explained once again by the fact that the database of “Club Hernie” comes from high-volume centres and surgeons, while the database of ISO-RAISIN only collects data from voluntary medical centres. This may favour highly specialized centres for groin hernia surgery as has previously been demonstrated in different studies worldwide [12]^1314^. Moreover, in the French report, the ISO-RAISIN data are recorded for both “groin” and “ventral” hernias which are completely different from only “groin hernias” which have different risk factors for SSIs.

Investigations of risk factors for SSI following GHR in the literature are sparse, and usually concentrates on patient-related predictive factors only. The study by Taylor et al. [15] in 2004 included data from 32 Scottish hospitals with 2,665 patients who had groin hernia surgery. Overall, 5.2% had SSI. The study pointed out after two multivariate analyses, two SSI risk factors: absence of antibiotic prophylaxis (*p*  =  0.002) and an NNIS (National nosocomial infections surveillance system) local score of 1 or 2 (*p*  =  0.021). The NNIS score is calculated by assigning one point to each of the following risk factors present: ASA score of 3 or more, operation classified as contaminated or dirty, and operation lasting less than 55 min, On the other hand, the study by Sereysky et al. [5], collected all the data of the American College of Surgeons National Surgical Quality Improvement Program (NSQIP). It included patients older than 18 years old with elective initial open inguinal hernia repair (thus excluding bilateral hernias, emergencies, unclean surgeries and operations conducted by laparoscopy). They reported SSI for 0.4% patients. After multiple logistic regression, only 3 factors (out of 17) were independently associated with SSIs: diabetes, BMI  ≥  35 kg/m^2^, and current smoking. In the latter study, antibiotic prophylaxis was not tracked. According to the HerniaSurg group, guidelines published in 2018, antibiotic prophylaxis is recommended in “open mesh repair in a high-risk environment” [3]. In our study, antibiotic prophylaxis was not used according to the previous guidelines and those of the French association of Anaesthesia (SFAR) until 2018. However, in 2018, SFAR guidelines changed and recommended antibiotic prophylaxis for mesh repair whatever the surgical approach. We can consider that between June 2018 (date of the 2018 SFAR guidelines) and May 2019 (end of our study), antibiotic prophylaxis in our study was used when recommended. Despite that, it seems evident that no difference exists between the two groups having benefited from a mesh repair whatever the surgical approach: or whether antibiotic prophylaxis was given. Furthermore, our study is different with 54% of patients operated laparoscopically and 16.5% with bilateral hernia repair. Our study is the only one to demonstrate the absence of difference in terms of SSI rates between open GHR and laparoscopic GHR, especially without antibiotic prophylaxis. This result is indirectly in accordance with the one of Köckerling et al. [6] where the benefit of antibiotics could only be limited at the open GHR because in fact they integrated emergency GHR in their analysis.

Concerning fixation method**,** our results seem similar to those of the review of Sanders et al. [16]. However, it is important to underline that this review encompassed twelve randomized clinical trials (majority of which were assessed as low or very low quality), including only 1992 primary open anterior inguinal hernia repairs. Four studies compared *n*-butyl-2 cyanoacrylate (NB2C) glues to sutures, two compared self-fixing meshes to sutures, four compared fibrin sealant to sutures, one compared tacks to sutures, and one compared absorbable sutures to non-absorbable sutures. No significant difference in surgical site infection rates between fixation methods were demonstrated.

Köckerling et al. [17] analyzed patients with inguinal hernia repair from the Herniamed registry. Those patients taking anticoagulants were significantly associated with higher deep infection rates, albeit for incisional hernias rather than GHR. In our study, that focused on GHR, treatment with anticoagulants was not associated with higher SSI rates.

## Limitations

The main limits of our study are respectively the involvement of French and high volumes centres only.

Moreover, the absence of significance regarding the type of mesh used and the risk of SSI, can be explained by the few numbers of SSIs: 57, despite a large number of patients in our cohort, more than 20,000. More precisely, due to the very low incidence of SSI (0.26%), despite the large number of cases in this series (21,976), the risk of a type II error must be considered. Indeed, for an exposure with an assumed prevalence of 50% in a sample, the statistical power was 80% to detect a risk ratio of 2.2 (corresponding to an incidence of SSI equal to 0.16% in unexposed persons and 0.36% in exposed persons). Although the lack of identification of risk factors for SSI could be due to limited statistical power, our cohort is one of the largest reported, which may suggest that a larger sample size would simply identify factors with weak association with SSI, and likely relatively low clinical relevance.

## Conclusion

In our large observational multicentre cohort of 25,593 groin hernia repairs in France, we did not find any patient or surgical risk factors that influenced the risk of SSI within a year of surgery. In particular there were no significant differences in SSI between laparoscopic and open surgery, or between different types of mesh. The overall rate of SSI was very low in our study that included patients from high volume centres, which suggests that the expected SSI rate in such a context is expected to be low.

### Electronic supplementary material

Below is the link to the electronic supplementary material.

Supplementary file 1 (DOCX 13 KB)

## Appendix 1

### Definitions

*Mesh characteristics

- Were defined according to Amid’s classification [8]: macroporous (prolene/polypropylene), microporous (ePTFE).
- The description of meshes as in « polyester», « polypropylene» « PTFE» corresponds to the main component of the mesh.
  More precisely, here, in our study
  - Non resorbable polyester and polypropylene meshes were considered as « macroporous».
  - Non resorbable PTFE meshes were considered as « microporous».
    Physical effort encompassed physical work and/or frequent sport (more than once per week).
    Dissection factors encompassed surgical history of
- Mac Burney.
- Other intraperitoneal surgery.
- Peritoneal vascular bypass.
- Transvesical adenomectomy.
- Homolateral iliac dissection.
- Radical prostatectomy.
- Other extra peritoneal surgery.
- Pelvic radiotherapy.

Recurrence: clinical or radiological presence of the same groin hernia (on the same side).

Smoking encompassed only daily smokers.

*Hernia characteristics

- Symptomatology of hernia: asymptomatic or symptomatic (with or without emergency criteria).
- Type of hernia: inguinal (direct or indirect), or femoral.
- Primary or recurrent.
- Uni or bilateral.

## Appendix 2

Centers for Disease Control criteria for definition of superficial surgical site infection.

Infection occurs within 30 days after operation and Infection involves only skin or subcutaneous tissue of the incision and at least one of the following

1. Purulent drainage, with or without laboratory confirmation from the superficial incision.
2. Organisms obtained from an aseptically obtained culture of fluid or tissue from the superficial incision.
3. At least one of the following signs or symptoms of infection: pain or tenderness, localized swelling, redness or heat and superficial incision is deliberately opened by surgeon, unless incision is culture negative.
4. Diagnosis of superficial incisional SSI by the surgeon or attending physician.

Do not report the following conditions as SSI

1. Stitch abscess (minimal inflammation and discharge confined to the points of suture penetration).
2. Infection of an episiotomy or newborn circumcision site.
3. Infected burn wound.
4. Incisional SSI that extends into the fascial and muscle layers.

Abdominal pressure

- Dysuria (>  2/night or weak stream or delay before urination).
- Constipation  >  3 days.
- Strength work (multi daily carrying of loads  > 10 kgs).
- Intensive sports activities.
- Ascite.

Physical effort

- Physical work and/or frequent sport (more than once per week).

Dissection factors: surgical history of

- Mac Burney.
- Other intraperitoneal surgery.
- Peritoneal vascular bypass.
- Transvesical adenomectomy.
- Homolateral iliac dissection.
- Radical prostatectomy.
- Other extra peritoneal surgery.
- Pelvic radiotherapy.

## Abbreviations

GHR: Groin hernia repair
SSI: Surgical site infection
