# Supplementary material for: Risk factors for surgical site infection after groin hernia repair: does the mesh or technique matter?
Source: Hernia. 2021 Oct 1;26(1):233–42. doi: 10.1007/s10029-021-02512-7 (PMC8881239; doi:10.1007/s10029-021-02512-7)
Supplement: Supplementary file 1 — (DOCX 13 KB) [file 10029_2021_2512_MOESM1_ESM.docx]

**Table S1: univariate analysis – risk factors of SSIs : sides with a mesh and surgical factors**

| **Surgical factors** | **Risk of SSI – sides with mesh** |
| --- | --- |
| **Recurrences** | p>0.99 |
| No | 48/22098 (0.22%) |
| Yes | 3/1631 (0.18%) |
| **Technique** | p=0.486 |
| laparoscopy | 26/13343 (0.19%) |
| Open | 26/10756 (0.24%) |
| **Mesh Type** | p=0.638 |
| Non resorbable polyester | 18/9678 (0.19%) |
| Non resorbable polypropylene | 33/13310 (0.25%) |
| Non resorbable Type undefined | 1/826 (0.12%) |
| Resorbable Synthetic | 0/7 (0.00%) |
| **Porosity** | p=0.406 |
| Microporous | 0/673 (0.00%) |
| Macroporous | 52/23148 (0.23%) |
| **Fixation** | p=0.402 |
| No | 26/13980 (0.19%) |
| Yes- Mixte | 7/3071 (0.23%) |
| Yes- Non resorbable | 7/1916 (0.37%) |
| Yes - Resorbable | 11/4953 (0.22%) |
| **Nerve preservation** | p>0.99 |
| No | 0/71 (0.00%) |
| Yes | 51/23760 (0.21%) |
| **Infiltration Naropeine** | p=0.189 |
| No | 14/8910 (0.16%) |
| Yes | 36/14822 (0.24%) |
| **Side** | p=0.783 |
| Left | 25/11134 (0.22%) |
| Right | 27/12965 (0.21%) |
